# Drug resistance testing through remote genotyping and predicted treatment options in human immunodeficiency virus type 1 infected Tanzanian subjects failing first or second line antiretroviral therapy

**DOI:** 10.1371/journal.pone.0178942

**Published:** 2017-06-05

**Authors:** Jenny Svärd, Sabina Mugusi, Doreen Mloka, Ujjwal Neogi, Genny Meini, Ferdinand Mugusi, Francesca Incardona, Maurizio Zazzi, Anders Sönnerborg

**Affiliations:** 1 Division of Clinical Microbiology, Department of Laboratory Medicine, Karolinska Institutet, Karolinska University Hospital, Stockholm, Sweden; 2 Department of Clinical Pharmacology, School of Medicine, Muhimbili University of Health and Allied Sciences, Dar es Salaam, Tanzania; 3 Department of Pharmaceutical Microbiology, Muhimbili University of Health and Allied Sciences, Dar es Salaam, Tanzania; 4 Department of Medical Biotechnology, University of Siena, Siena, Italy; 5 Department of Internal Medicine, Muhimbili University of Health and Allied Sciences, Dar es Salaam, Tanzania; 6 EuResist Network GEIE, Roma, Italy; 7 Unit of Infectious Diseases, Department of Medicine Huddinge, Karolinska Institutet, Karolinska University Hospital, Stockholm, Sweden; University of Pittsburgh, UNITED STATES

## Abstract

**Introduction:**

Antiretroviral therapy (ART) has been successfully introduced in low-middle income countries. However an increasing rate of ART failure with resistant virus is reported. We therefore described the pattern of drug resistance mutations at antiretroviral treatment (ART) failure in a real-life Tanzanian setting using the remote genotyping procedure and thereafter predicted future treatment options using rule-based algorithm and the EuResist bioinformatics predictive engine. According to national guidelines, the default first-line regimen is tenofovir + lamivudine + efavirenz, but variations including nevirapine, stavudine or emtricitabine can be considered. If failure on first-line ART occurs, a combination of two nucleoside reverse transcriptase inhibitors (NRTIs) and boosted lopinavir or atazanavir is recommended.

**Materials and methods:**

Plasma was obtained from subjects with first (n = 174) or second-line (n = 99) treatment failure, as defined by clinical or immunological criteria, as well as from a control group of ART naïve subjects (n = 17) in Dar es Salaam, Tanzania. Amplification of the *pol* region was performed locally and the amplified DNA fragment was sent to Sweden for sequencing (split genotyping procedure). The therapeutic options after failure were assessed by the genotypic sensitivity score and the EuResist predictive engine. Viral load was quantified in a subset of subjects with second-line failure (n = 52).

**Results:**

The HIV-1 *pol* region was successfully amplified from 55/174 (32%) and 28/99 (28%) subjects with first- or second-line failure, respectively, and 14/17 (82%) ART-naïve individuals. HIV-1 *pol* sequence was obtained in 82 of these 97 cases (84.5%). Undetectable or very low (<2.6 log_10_ copies/10^−3^ L) viral load explained 19 out of 25 (76%) amplification failures in subjects at second-line ART failure. At first and second line failure, extensive accumulation of NRTI (88% and 73%, respectively) and NNRTI (93% and 73%, respectively) DRMs but a limited number of PI DRMs (11% at second line failure) was observed. First line failure subjects displayed a high degree of cross-resistance to second-generation NNRTIs etravirine (ETR; 51% intermediate and 9% resistant) and rilpivirine (RPV; 12% intermediate and 58% resistant), and to abacavir (ABC; 49% resistant) which is reserved for second line therapy in Tanzania. The predicted probability of success with the best salvage regimen at second-line failure decreased from 93.9% to 78.7% when restricting access to the NRTIs, NNRTIs and PIs currently available in Tanzania compared to when including all approved drugs.

**Discussion:**

The split genotyping procedure is potential tool to analyse drug resistance in Tanzania but the sensitivity should be evaluated further. The lack of viral load monitoring likely results in a high false positive rate of treatment failures, unnecessary therapy switches and massive accumulation of NRTI and NNRTI mutations. The introduction of regular virological monitoring should be prioritized and integrated with drug resistance studies in resource limited settings.

## Introduction

In 2015, more than 500 000 human immunodeficiency virus (HIV) infected subjects in low-middle income countries received second-line antiretroviral therapy (ART). A recent modelling study estimated that the number of subjects on second-line ART will increase substantially, and by 2020 between 0.5 million and 3 million people in sub-Saharan Africa (SSA) will need second line therapy [1]. The World Health Organization (WHO) recommends a regimen of a ritonavir-boosted protease inhibitor (PI/r) plus two nucleoside or nucleotide reverse transcriptase inhibitors (NRTI) for second-line ART in SSA and this approach is becoming increasingly common [2, 3]. Studies so far have demonstrated sustained viral suppression for up to two years among the majority of adult [2, 3] and paediatric [4] subjects undergoing second-line treatment following first-line failure in SSA. However, ART failure and the presence of major PI mutations have been reported in a minority of South Africans given PI/r containing second-line ART [5].

In Tanzania, ART has been freely available since 2004. In 2015, the prevalence of HIV-1 infection was 4.7% among adults and the ART coverage was estimated to be 53% [6]. According to national guidelines [7], the default first-line regimen (given to HIV-1-infected subjects at WHO stage 3 and 4 or with CD4 < 500 cells/10^−6^ L) is tenofovir (TDF) + lamivudine (3TC) + efavirenz (EFV) although the NRTIs may be changed to other available options such as zidovudine (AZT) or emtricitabine (FTC) and EFV may be replaced by nevirapine (NVP) under certain conditions. Treatment failure is identified using clinical and immunological parameters while virological monitoring, although recommended, is not part of routine clinical practice. If failure on first-line ART occurs, a combination of two NRTIs (including TDF, AZT or abacavir [ABC] depending on first line regimen) and boosted lopinavir (LPV/r) or atazanavir (ATV/r) is recommended. However, in case of second-line ART failure no third-line regimen is described in the national guidelines [7] and few drug options remain—no PIs other than lopinavir and atazanavir, or drugs of newer classes such as entry inhibitors or integrase inhibitors, are currently available in Tanzania.

This cross-sectional study aimed to describe the viral genotypic pattern at treatment failure of first-line ART as well as second-line PI/r-based ART in a real-life Tanzanian setting using the established clinical and immunological failure (CIF) criteria. Since drug genotypic resistance testing (GRT) is not readily available in SSA, we used the “split genotyping procedure” [8], where PCR amplification is performed locally while sequencing is carried out at a remote reference laboratory. Drug susceptibility was inferred by rule-based genotypic sensitivity score (GSS) algorithm and the EuResist bioinformatics predictive engine was used to predict the probability of success of future treatment options [9].

## Materials and methods

### Ethical approval

Ethical approval for carrying out the study was obtained from the Senate Research and Publications Committee at the Muhimbili University of Health and Allied Sciences (Ref. No. MU/01/1022/0122/10). Written informed consent was obtained from the subjects included in the study. All participants were given a copy of the signed consent form, which contained information about the study and contact details of the principal investigator and the ethics review board, both in English and Kiswahili.

### Subjects

Subjects with suspected first or second line treatment failure were approached consecutively at two clinics; Muhimbili National Hospital and Amtulabhai Karimjee Treatment failure Clinic in Dar es Salaam, Tanzania, between June 2009 and September 2014. As viral load testing is not part of clinical practice in Tanzania, identification of subjects with ART failure was based on CIF only [10]. WHO guidelines for a public health approach to ART define immunological failures as: a CD4 count decrease to below baseline in the absence of concurrent infections, a decrease of more than 50% from the peak value, or persistent CD4 below 100 cells/mm^3^ while on treatment [10]. One-hundred-seventy-four consenting subjects with first-line ART failure and 99 consenting subjects with second-line ART failure were recruited. The sample size was obtained based on what was possible to access considering time and resources available. In addition, 17 treatment naïve HIV-1 infected subjects from the Amtulabhai Karimjee Treatment failure Clinic who consented were included in the study as a control group (to test the split genotyping procedure using patient samples likely to have high viral loads and also to compare drug resistance mutation [DRM] profiles). Demographical and clinical information, ART exposure (dates and drug combinations), WHO stage and CD4 counts were extracted from patient charts.

CD4 cells were determined by routine flow cytometry at the Muhimbili National Hospital. Plasma from a subgroup of 52 subjects with CIF on second line PI/r-based therapy, of which 27 were PCR positive and 25 were PCR negative, were tested for HIV-RNA using COBAS AmpliPrep/Cobas TaqMan HIV-1 test v 2.0 (CAP/CTM 48) (Roche Diagnostics, Basel, Switzerland) at the National Health Laboratory Quality and Training Centre, Dar es Salaam.

### Genotypic resistance testing (GRT)

GRT was achieved using the previously described split genotyping procedure [8] in which the first steps of GRT is performed at a local laboratory and the second steps at a centralized laboratory. RNA was thus extracted from plasma locally at Muhimbili University of Health and Allied Sciences (MUHAS) by the QIAamp Viral RNA Mini Kit (Qiagen, Sollentuna, Sweden), followed by reverse-transcription PCR (RT-PCR) to generate cDNA using ImProm-II Reverse Transcription System (Promega Biotech, Nacka, Sweden), according to the manufacturer’s instructions. Nested *pol* gene amplification was carried out according to EuResist guidelines, as described elsewhere [11] and visualized by gel electrophoresis. The PCR products were purified using QIAquick PCR Purification Kit (Qiagen). Thereafter, the purified PCR products were sent to the Division of Clinical Microbiology, Karolinska Institutet, Stockholm, Sweden, and subjected to bi-directional population sequencing.

Sequences were manually scrutinized and edited using Geneious R6.1.6 (Biomatters, Auckland, New Zealand). Sequences with full length protease gene (PR; 1–99 aa) and partial reverse transcriptase gene (RT; 1–230) were used for further analysis. For positions where a second peak was observed with peak height ≥50% of the main peak, a wobble nucleotide was assigned. The sequences were deposited to GenBank with accession numbers KX775225-KX775306.

HIV-1 subtyping was performed with REGA version 3.02 [12] followed by phylogenetic tree analysis with reference sequences in PhyML [13]. The reference sequences were downloaded from Los Alamos database (www.hiv.lanl.gov). Multiple sequence analysis was performed in ClustalW algorithm incorporated in Geneious R6.1.6. The alignment file was used for phylogenetic analysis by maximum likelihood method in PhyML with the general time reversible plus gamma substitution model (GTR+G) as best fitted model identified by the FindModel tool (http://www.hiv.lanl.gov/content/sequence/findmodel/findmodel.html). The approximate Likelihood Ratio Test (aLRT) was applied for branch support and the tree consisting of only Tanzanian sequences was visualized using FigTree (http://tree.bio.ed.ac.uk/software/figtree/). For clustering analysis, 5–7 sequences per cluster plus 3 outliers were submitted to the HIV-1 BLAST tool. The top three hits per sequence were downloaded and aligned to generate a new phylogenetic tree as described above. The presence of drug resistance mutations was assessed by using the Stanford HIVdB online tool [14].

### Genotypic sensitivity scores (GSS) and prediction of optimal future therapy

For assessment of GSS for current and future drug options, Rega version 9.1.0 [15] was employed. Also, the EuResist Prediction Engine (http://engine.euresist.org/) was used for prediction of the optimal salvage treatment regimen, based on the genotype at failure.

### Statistics

Data were analysed using SPSS Statistics 22 (IBM Corporation, Armonk NY, USA) and graphs were generated with the aid of GraphPad Prism 5 (GraphPad Software, La Jolla CA, USA). Descriptive analysis was performed: mean or median and interquartile range (IQR) was used for summarising continuous variables, and frequencies and percentages for categorical variables. A p-value < 0.05 was considered significant. For analyses examining the correlation between viral load and CD4, and between viral load and treatment duration, nonparametric correlation analysis (Spearman’s rho, two-tailed) was used in SPSS.

## Results

### PCR amplification outcome, sample population and viral load test results

Using the split genotyping procedure, 14 of 17 (82%) samples from ART-naïve individuals were successfully amplified, of which 13 yielded *pol* gene sequences suitable for further analysis. For first-line and second-line failures respectively, as defined by CIF criteria, only 55 of 174 (32%) and 28 of 99 (28%) samples were successfully amplified, with corresponding 43 and 26 sequences generated. Clinical information about the subjects for whom sequencing succeeded is given in Table 1 (see also S1 File). All subjects were above 18 years old except for five first line failure subjects (aged 5, 7, 14, 16 and 17 respectively) and one second line failure patient (aged 15).

**Table 1:** Demographics of subjects with successfully obtained HIV-1 *pol* sequences.

Characteristic	ART-naïve (n = 13)	1^st^ line failure (n = 43)	2^nd^ line failure (n = 26)
**Age** median years (IQR)	33 (28, 36)	38 (33, 48)	39 (31, 46)
Range	25–59	5–63	15–78
*missing*, *n (%)*	*0 (0*.*0%)*	*1 (2*.*3%)*	*0 (0*.*0%)*
**Gender** % Female	100%	60.5%	73.1%
*missing*, *n (%)*	*0 (0*.*0%)*	*0 (0*.*0%)*	*0 (0*.*0%)*
**Time since diagnosis** median years (IQR)	3.5 (0.03, 5.6)	4.7 (3.4, 6.3)	6.7 (4.9, 7.2)
*missing*, *n (%)*	*0 (0*.*0%)*	*6 (13*.*0%)*	*6 (23*.*1%)*
**CD4 at start of ART** median cells/μl (IQR)	N.A.	50 (11, 175)	109 (28, 180)
*missing*, *n (%)*		*6 (13*.*0%)*	*2 (7*.*7%)*
**CD4 at sampling** median cells/μl (IQR)	445 (147, 579)	24 (7, 57)	93 (32, 141)
*missing*, *n (%)*		*7 (16*.*3%)*	*2 (7*.*7%)*
**Viral load** log_10_ copies/mL median (IQR)	N.A.	N.A.	4.6 (4.0, 5.2)
*missing*, *n (%)*			*1 (3*.*8%)*
**WHO stage at sampling** n (%)			
I	7 (53.8%)	0 (-)	1 (3.8%)
II	4 (30.8%)	9 (20.9%)	8 (30.8%)
III	1 (7.7%)	14 (32.6%)	11 (42.3%)
IV	1 (7.7%)	17 (39.5%)	4 (15.0%)
*Missing*	*0 (-)*	*3 (7*.*0%)*	*2 (7*.*7%)*
**ART duration** months mean (IQR)			
1^st^ line therapy	N.A.	49.1 (37.1, 56.7)	63.4 (40.7, 74.5)
*missing*, *n (%)*		*5 (11*.*6%)*	*3 (11*.*5%)*
2^nd^ line therapy	N.A.	N.A.	17.7 (2.9, 31.4)
*missing*, *n (%)*			*3 (11*.*5%)*
**ART initiated** n (%)	N.A.		
*1*^*st*^ *line regimen*			
d4T + 3TC + NVP		19 (44.2%)	11 (42.3%)
AZT + 3TC + NVP		11 (25.6%)	7 (26.9%)
AZT + 3TC + EFV		8 (18.6%)	4 (15.4%)
TDF + FTC/3TC + EFV		3 (7.0%)	0 (-)
d4T + 3TC + EFV		0 (-)	3 (15.4%)
*Missing*		*2 (4*.*7%)*	*1 (3*.*8%)*
*2*^*nd*^ *line regimen*			
TDF + FTC + LPV/r			19 (73.1%)
ABC + ddI + LPV/r			3 (11.5%)
ABC + 3TC +LPV/r			1 (3.8%)
TDF + FTC +ATV/r			1 (3.8%)
*Missing*			*2 (7*.*7%)*

ART = antiretroviral therapy, d4T = stavudine, 3TC = lamivudine, NVP = nevirapine, AZT = zidovudine, EFV = efavirenz, TDF = tenofovir disoproxil fumarate, FTC = emtricitabine, LPV/r = ritonavir-boosted lopinavir, ABC = abacavir, ddI = didanosine, ATV/r = ritonavir-boosted atazanavir.

To determine whether the low rate of amplification success was due to failure of the methodology or due to misclassification of treatment failure by the CIF criteria, viral load testing was performed in 52 subjects having second-line CIF for whom residual plasma was available, of which 27 had successfully amplified HIV-1 *pol* region while for the remaining 25 PCR amplification had been unsuccessful despite decreasing CD4 counts indicating treatment failure. The results revealed that for the majority of second-line samples for which PCR amplification was unsuccessful (n = 25), HIV-RNA levels were below the limit of quantification (target not detected; n = 2 or <1.3 log_10_ copies/10^−3^ L; n = 4) or very low (1.3–2.6 log_10_ copies/10^−3^ L; n = 13) or low (2.6–3 log_10_ copies/10^−3^ L; n = 3). Only three samples (12%) were > 3 log_10_ copies/10^−3^ L (3.27 log_10_ copies/10^−3^ L; 4.74 log_10_ copies/10^−3^ L and 5.54 log_10_ copies/10^−3^ L). Consequently, among the samples tested for viral load, PCR amplification was successful in 27/30 (90%) of the samples with a viral load of >3 log_10_ copies/10^−3^ L. Among the PCR-positive second-line failure samples (n = 27), the viral load ranged between 3.06 and 5.74 log_10_ copies/10^−3^ L. For the 26 samples which generated usable sequences and were included in the genotypic analysis, the median viral load was 4.8 log_10_ copies/10^−3^ L (IQR 4.0, 5.4). There was no significant correlation between viral load and CD4 at time of failure. Neither was there a correlation between viral load and treatment duration (Spearman’s rho, two-tailed).

### Drug resistance mutations at first- and second-line failures

**Fig 1:**
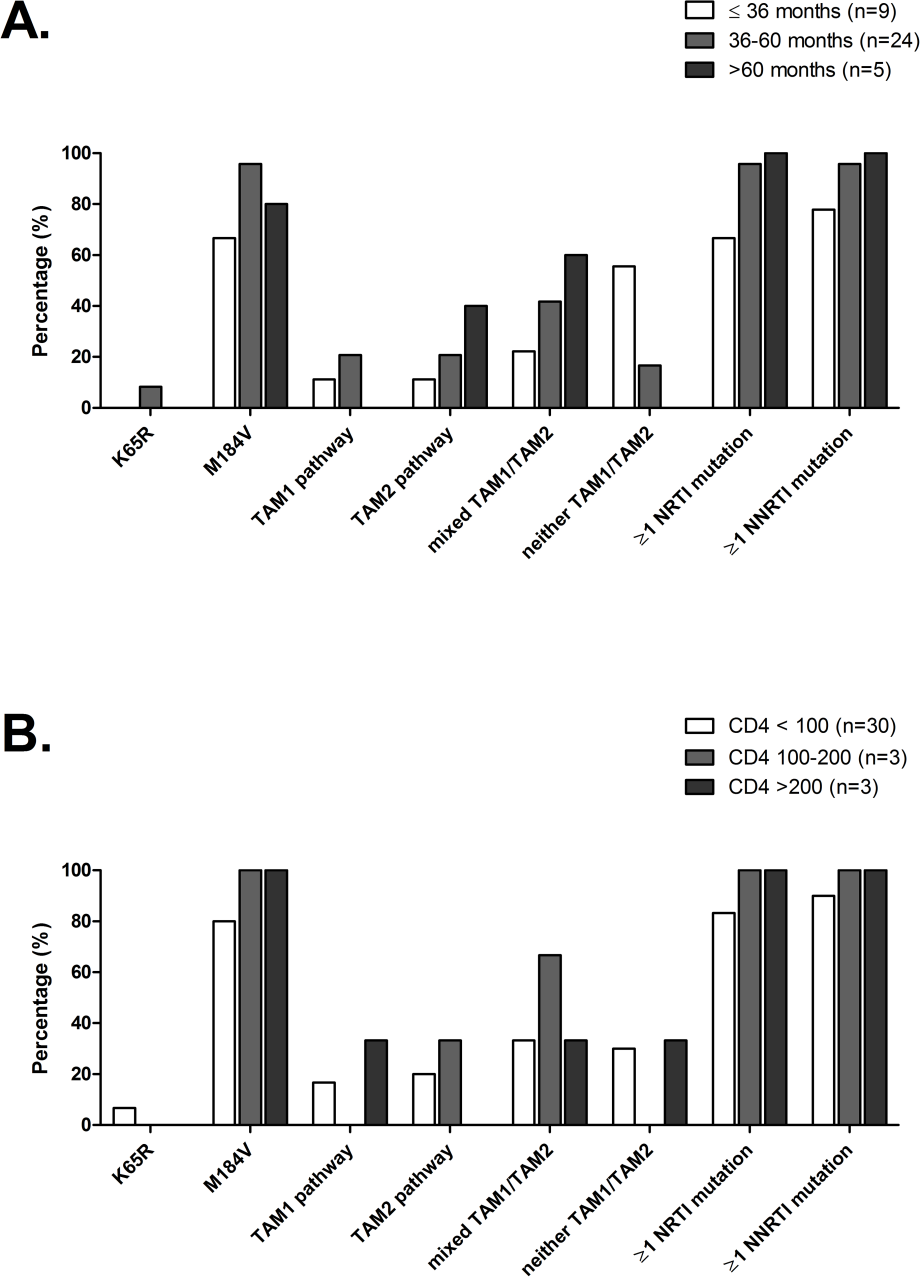
Antiretroviral drug resistance mutations in Tanzanian subjects at first line clinico-immunological ART failure. A) stratified by treatment duration (n = 38); B) stratified by CD4 cells at failure (n = 36).

Among 43 first-line failing subjects from whom a *pol* sequence was obtained, almost all (37/43; 86%) had ≥1 NNRTI mutation as well as ≥1 NRTI mutation. The proportion of subjects with NRTI and NNRTI mutations increased with the duration of ART. For those treated <36 months (n = 9 out of 38 subjects with data on ART duration), six subjects (67%) had NRTI mutations and seven subjects (78%) had NNRTI mutations (one patient had NNRTI mutations only and six had both NRTI and NNRTI mutations). For those treated 36–60 months (n = 24), 23 of 24 subjects (96%) had NRTI mutations and similarly 23 of 24 subjects (96%) had NNRTI mutations (100% had either NRTI and/or NNRTI mutations: NRTI only: 1 (4%); NNRTI only: 1 (4%); both NRTI and NNRTI: 22 (95%)). For the five subjects treated >60 months, all subjects had both NRTI and NNRTI mutations (Fig 1). For comparison, out of the 13 treatment naïve subjects, one displayed the K103N mutation and one the E138A mutation.

Only three out of 26 (11.5%) subjects failing PI/r-based second-line ART with detectable viral load and a readable sequence had major PI mutations (V82A; V32I + I47A; M46I + I54V + V82A). From a public health point of view in a setting without access to viral load testing, the rate of major PI mutation was only 3% of the 99 subjects considered to have second-line ART failure, according to CIF criteria. Among the 26 subjects failing second-line therapy, 17 (65%) had both NRTI and NNRTI mutations and 4 (15%) had either NRTI or NNRTI mutations.

### Assessment of future options after therapy failure

**Fig 2:**
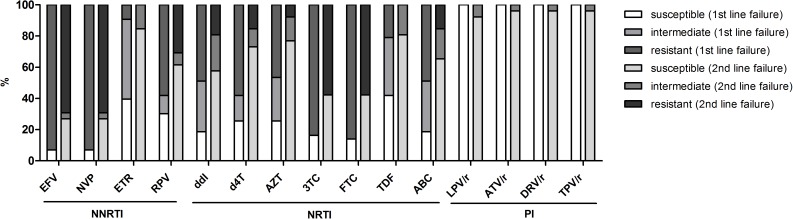
Genotypic sensitivity scores for all reverse transcriptase and protease inhibitors. Percentage (%) of patient strains classified as susceptible, intermediate or resistant as per the Rega V.9.1.0 algorithm; first line failure (n = 43) and second line virological failure cases (n = 26) presented with separate paired bars (first line left bar, second line right bar).

Among first-line ART failures, there was a widespread resistance to the first generation NNRTI, efavirenz and nevirapine (93% resistant for both drugs), and a high degree of cross-resistance to second-generation NNRTIs etravirine (ETR; 51% intermediate and 9% resistant) and rilpivirine (RPV; 12% intermediate and 58% resistant). Forty-nine percent (49%) of first-line failures also displayed cross-resistance to ABC, which in general is reserved for second line therapy in Tanzania. Second line failure subjects displayed somewhat lower resistance to EFV and NVP (69% resistance for both drugs) and lower cross-resistance to ETR (15% intermediate and 0% resistant) and RPV (8% intermediate and 31% resistant), while resistance to FTC and 3TC was high (58% resistant for both drugs). (Fig 2).

**Fig 3:**
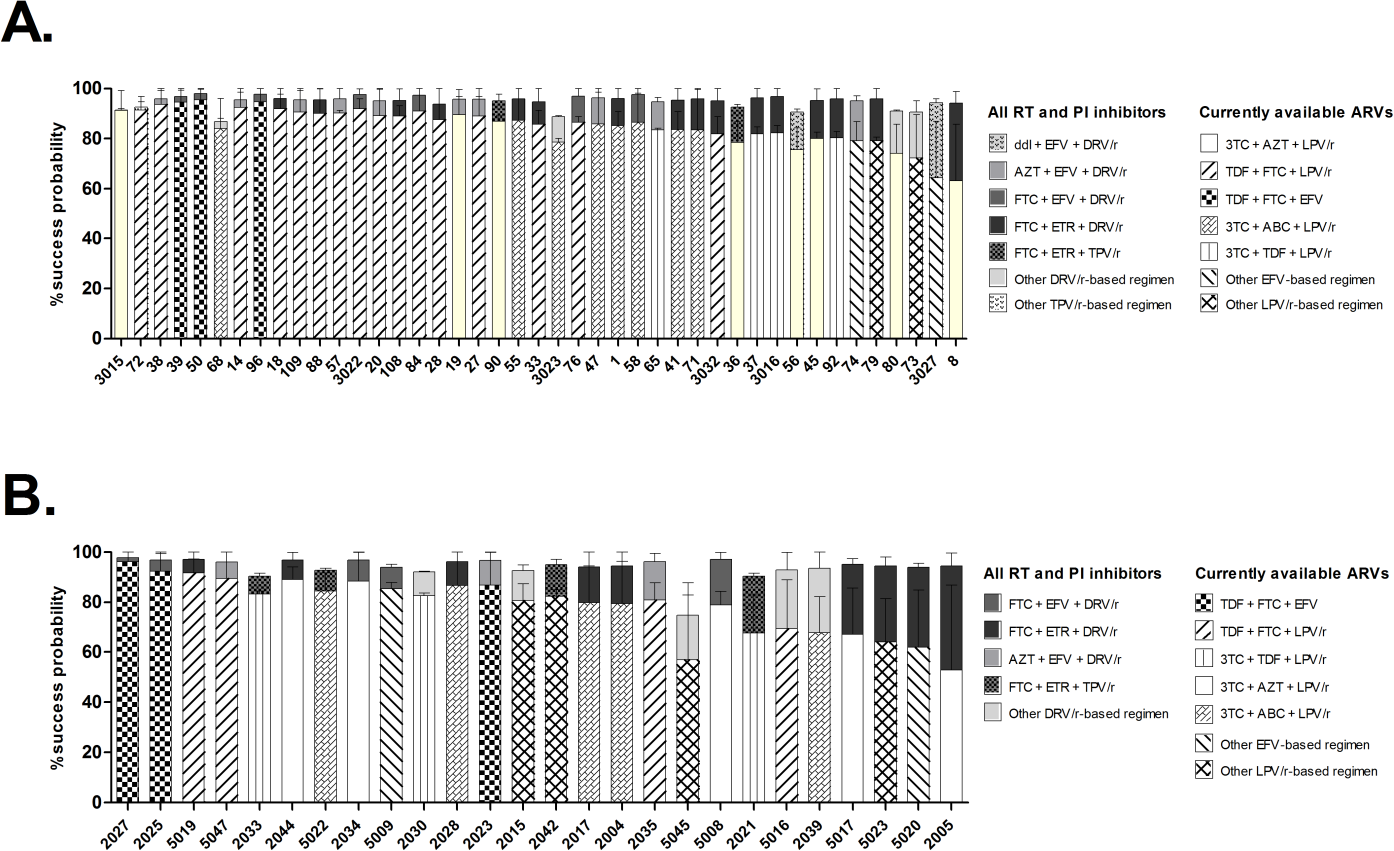
Success probability of future regimen by the EuResist prediction engine. Prediction is based on drug resistance mutation profile and data on previous drug exposure, age, gender and CD4 at failure. Results are presented in ordered of increasing difference between a scenario where all approved NRTIs, NNRTIs and PIs are available versus currently available drugs in Tanzania. A) subjects failing first line therapy; B) subjects failing second line therapy (viral load included in prediction model).

Future therapy options were assessed by the GSS approach (Fig 2) and by the EuResist prediction engine (Fig 3). For the currently most common second-line regimen in Tanzania (TDF + FTC + LPV/r), the combined median GSS was 2.0 (IQR 2.0, 2.5) for all first-line failure subjects, corresponding to two active drugs, which is the minimum target GSS for treatment-experienced subjects with limited treatment options (https://rega.kuleuven.be/cev/avd/files/software/rega_algorithm/Rega_HIV1_Rules_v9.1.0.pdf).

The success probability of the predicted optimal therapy was also investigated using the EuResist prediction engine for all first-line and second-line failure subjects, given their individual DRM profile. Firstly, when considering the TDF + FTC + LPV/r combination (the most widely used second line antiretroviral regimen in Tanzania), the median success probability was 87.7% (range 64.2–94.2%) for 39 out of the 43 first-line failure subjects. For the remaining four subjects, this combination was not among the top ten recommended ART regimens (and hence no estimated success probability was given). Two of these subjects had DRM patterns which confer resistance to FTC according to the REGA criteria, and one of them additionally displayed intermediate resistance to TDF [15]. Secondly, two hypothetical scenarios were compared: 1) all approved NRTIs, NNRTIs and PIs were available for building the treatment regimen, and 2) only currently available antiretrovirals in Tanzania were included in the optimal future regimen (ABC, FTC, didanosine (ddI), 3TC, d4T, TDF, AZT, EFV, NVP, ATV/r, LPV/r). Among the first line failure cases, the mean success probability was reduced from 94.9% to 85.0% (reduction range 0.2–31.3%) when restricting antiretroviral access to the current situation in Tanzania (Fig 3A). For the second-line failure subjects, the corresponding mean reduction was from 93.9% to 78.7% (reduction range 1.7–41.2%) (Fig 3B).

### Molecular epidemiology of HIV-1 subtypes

The subtype analysis identified 41% subtype A1, 27% subtype C, 9% subtype D, 5% CRF10_CD, 17% recombinant (A1C, A1D or other D recombinants), and 1% unclassified. Subtype A1 *pol* sequences primarily clustered with other sequences from Tanzania, Kenya and Uganda (data not shown). Subtype D *pol* sequences clustered with other sequences from Tanzania and Uganda. Subtype C *pol* sequences clustered together with *pol* sequences from Tanzania, South Africa, Zambia and Botswana (9 sequences) or with *pol* sequences from Ethiopia (7 sequences).

## Discussion

Despite the increasing use of PI-based second-line ART in SSA, little is known about the real-life treatment outcomes, DRM patterns at therapy failure, and further treatment options thereafter. As viral load monitoring is not yet part of routine clinical practice in Tanzania, treatment failure is defined by clinico-immunological criteria. The study therefore evaluated the consequences of using these criteria for the definition of ART failure and the assessment of drug resistance. Also, since access to point of care (POC) diagnostics is lacking also for GRT all over the world [16], the split genotyping procedure for assessment of DRM was evaluated.

In pilot evaluations, the “split genotyping procedure” has shown that laboratory technicians without or with limited background in molecular diagnostics can be successfully trained to generate HIV DNA fragments to be shipped and sequenced remotely with satisfactory results [8], although this needs further validation including viral load verification given the high PCR failure rates. In the present study, the first part of the split GRT design was run at MUHAS (Dar es Salaam, Tanzania) and the second part at Karolinska Institutet (Stockholm, Sweden). The sequencing at Karolinska Institutet was successful in a large number of samples (82/97; 84.5%) for which a HIV DNA fragment was obtained at MUHAS, despite that a high degree of viral heterogeneity at the subtype level was found (subtypes A1, C, D, CRF10_CD, CRF A1C, A1D, other D recombinants and unclassified). Viral heterogeneity and spurious PCR products generating overlapping sequences could be explanatory factors behind sequencing failure despite successful PCR amplification (n = 15).

Although we did not perform any formal analysis of the sensitivity of the approach, the split design is likely to be a valid alternative to e.g. GRT on dried blood spots or other approaches of POC diagnostics for GRT [16]. We recognize that there might also be some additional issues related to sending amplified non-infectious proviral DNA through air-flights but according to our experience at the present global situation, the costs is low and the material is not considered to be a biohazard. Also, we showed that it can be used to describe the subtype distribution in a region, at least when a fragment of sufficient length is used.

The HIV-1 *pol* gene amplification success rate at MUHAS was however very low when we tested a large number of plasma samples from subjects who had been defined as first-line ART failures (success rate 38%) or second-line PI/r-based ART failures (success rate 28%) by clinico-immunological assessment in real-life settings by Tanzanian physicians. When we determined the viral load in a subset of the second-line failures (around half of which had successful HIV-1 *pol* amplification and around half of which had unsuccessful HIV-1 *pol* amplification) by quantitative PCR it was found that almost all subjects for whom the HIV-1 *pol* amplification had failed, had undetectable or very low viral load. In contrast, most (27/30; 90%) of the subjects with a viral load >3 log_10_ copies/10^−3^ L had amplification success. It can be argued that the local PCR failed in three subjects with a viral load of 2.6–3 log_10_ copies/10^−3^ L, which in a high-income country would be a potential problem. However, it is important to emphasize that WHO recommends switch to a new regimen when the viral load is > 1000 copies/ml (3 log_10_ copies/10^−3^ L) since the risk of HIV transmission is very low below this threshold[10], and that frequently a viral load between 2.6–3 log_10_ copies/10^−3^ L can be handled through increased adherence support[17], avoiding unnecessary switches in a situation with few therapeutic alternatives. Although we did not test the viral load in our first-line failures it seems likely that a similar pattern was the major cause of the amplification failures. These subjects may thus have been unnecessarily switched from an efficient ART regimen. Other recent studies from Tanzania [18, 19], Kenya [20] as well as a multi-center study across six SSA countries [21] demonstrated poor correlation between immunological and virological failure. For example, Sigaloff *et al*. showed that 46.9% of subjects who were defined as treatment failures based on the clinico-immunological criteria were found to have an HIV RNA level <1000 copies/10^−3^ L (<3 log_10_ copies/10^−3^ L) [21]. In our study the proportion was similar; 42%.

Furthermore, the subjects with the longest duration on a failing first line regimen in our study had accumulated the highest number of DRMs. Other studies have demonstrated that absence of virological monitoring and a delayed switch of therapy lead to a high degree of accumulation of DRMs [22] and an increased mortality [23]. Since 2013, WHO have included virological testing as the preferred monitoring strategy [10], which may eventually enable affordable viral load testing in resource-limited settings and shift the cost/benefit balance in favour of implementing virological monitoring on a broader scale. In addition, improved access to GRT, e.g. through the split genotyping procedure or other POC tests could inform on the best future regimens once treatment failure has been confirmed by viral load testing.

In our cohort of 26 HIV-1 infected subjects with virologically confirmed failure on second-line therapy, we detected major PI mutations in 11.5% (three individuals), which is the first report on emergence of major PI mutations in Tanzania. This data mirrors a larger study including 490 subjects receiving LPV/r in South Africa where the prevalence was 11% [5]. However it must be noted that the South African study included 44% of children (<15 years of age) and this may have contributed to the prevalence described.

In order to evaluate the remaining treatment possibilities for the failing subjects, we analysed the GSS as well as predicted the remaining options by the EuResist predictive engine [9]. Notably using GSS, 49% of first-line failure cases displayed resistance to ABC and 21% to TDF, which are used together with LPV/r as the standard second-line regimen in Tanzania. Additionally, more than half of first-line failure subjects and 15% of second-line failure subjects carried virus classified as intermediate resistant or resistant to ETR, while the level of resistance to RPV was even higher. The results show thus the potential value of PI/r inhibitors in second- and third-line ART in Tanzania as well as second-generation integrase inhibitors in the future.

The probability of success based on a predicted optimal therapy was also investigated using the EuResist prediction engine. Despite the high burden of NRTI mutations, when considering the TDF + FTC + LPV/r regimen the median success probability was as high as 87.7% (range 64.2–94.2%) for 39 of the 43 first-line failure subjects. These results are in line with the finding of the EARNEST trial, in which NRTIs retained substantial virological activity when given with a PI in second-line therapy [2]. It must be noted however that predictions of optimal future regimen demonstrated a mean reduction of success probability among second-line failure subjects from 93.9% to 78.7% when the choices were restricted to the NRTIs, NNRTIs and PIs currently available in Tanzania compared to when including all approved drugs of these categories. A limitation of this analysis was allowing 3-class regimens, which are uncommonly used in the clinical setting.

In summary, our study underscores the importance of using viral load for monitoring ART in resource-limited settings since the clinico-immunological monitoring in our study resulted in a very high frequency of unnecessary ART switches but also to an accumulation of extensive NRTI and NNRTI resistance in subjects failing with undetected viremia. When viral load is introduced for monitoring ART in these settings, our split genotyping procedure could be a valid and simple method for monitoring drug resistance to present and future antiretroviral agents although further validation is required to address potential PCR amplification issues. If a viral sequence can be obtained, bioinformatics methods can help the physicians to optimize the ARV regimens, especially for third-line ART in settings with limited therapeutic options.

## Supporting information

S1 File Demographic, clinical and genotype information of all study subjects. Age, gender, HIV-1 subtype, years since diagnosis, WHO stage, CD4 count, treatment regimen(s), treatment duration, optimal regimen and success probability predicted by EuResist prediction engine, genotypic sensitivity scores and HIV-1 drug resistance mutations are shown for 1^st^ line ART failure subjects, 2^nd^ line ART failure subjects and treatment naïve subjects.
(XLSX)

## References

[1] Estill J, Ford N, Salazar-Vizcaya L, Haas AD, Blaser N, Habiyambere V, et al The need for second-line antiretroviral therapy in adults in sub-Saharan Africa up to 2030: a mathematical modelling study. Lancet HIV. 2016 3;3(3):e132–9. doi: 10.1016/S2352-3018(16)00016-3 26939736 10.1016/S2352-3018(16)00016-3 PMC5688234

[2] Paton NI, Kityo C, Hoppe A, Reid A, Kambugu A, Lugemwa A, et al Assessment of second-line antiretroviral regimens for HIV therapy in Africa. N Engl J Med. 2014 7 17;371(3):234–47. doi: 10.1056/NEJMoa1311274 25014688 10.1056/NEJMoa1311274

[3] Schoffelen AF, Wensing AM, Tempelman HA, Geelen SP, Hoepelman AI, Barth RE. Sustained virological response on second-line antiretroviral therapy following virological failure in HIV-infected patients in rural South Africa. PLoS One. 2013;8(3):e58526 doi: 10.1371/journal.pone.0058526 23505529 10.1371/journal.pone.0058526 PMC3594302

[4] Dow DE, Shayo AM, Cunningham CK, Reddy EA. Durability of antiretroviral therapy and predictors of virologic failure among perinatally HIV-infected children in Tanzania: a four-year follow-up. BMC Infect Dis. 2014;14:567 doi: 10.1186/s12879-014-0567-3 25373425 10.1186/s12879-014-0567-3 PMC4225040

[5] Van Zyl GU, Liu TF, Claassen M, Engelbrecht S, de Oliveira T, Preiser W, et al Trends in Genotypic HIV-1 Antiretroviral Resistance between 2006 and 2012 in South African Patients Receiving First- and Second-Line Antiretroviral Treatment Regimens. PLoS One. 2013;8(6):e67188 doi: 10.1371/journal.pone.0067188 23840622 10.1371/journal.pone.0067188 PMC3694021

[6] Prevention Gap Report. UNAIDS, 2016. Available from: http://www.unaids.org/sites/default/files/media_asset/2016-prevention-gap-report_en.pdf

[7] National Guidelines for the Management of HIV and AIDS (5th Ed). In: The United Republic of Tanzania MoHaSW, editor. May 2015.

[8] Zazzi MM, G.; Emary, A.; Fikrie, N.; Kadima, S.; Marandu, E.; Mloka, D.; Mugusi, S.; Mutuku, S.; Njogu, W.; Worku, A., Bakari, M.; Fekade, D.; Mugusi, F.; Mwau, M.; Toti, M.; Incardona, F.; Sönnerborg, A. Training and strategy for HIV drug resistance testing in Eastern Africa. Sixth EDCTP Forum—Strengthening Research Partnership for Better Health and Sustainable Development; 9–12 October; Addis Ababa, Ethiopia 2011.

[9] Rosen-Zvi M, Altmann A, Prosperi M, Aharoni E, Neuvirth H, Sonnerborg A, et al Selecting anti-HIV therapies based on a variety of genomic and clinical factors. Bioinformatics. 2008 7 1;24(13):i399–406. doi: 10.1093/bioinformatics/btn141 18586740 10.1093/bioinformatics/btn141 PMC2718619

[10] Consolidated guidelines on the use of antiretroviral drugs for treating and preventing HIV infection—Recommendations for a public health approach WHO, 6 2013 Available from: http://www.who.int/hiv/pub/guidelines/arv2013/en/24716260

[11] EuResist Training Tools [23 April 2017]. Available from: https://www.euresist.org/training-tools.

[12] Pineda-Pena AC, Faria NR, Imbrechts S, Libin P, Abecasis AB, Deforche K, et al Automated subtyping of HIV-1 genetic sequences for clinical and surveillance purposes: performance evaluation of the new REGA version 3 and seven other tools. Infect Genet Evol. 2013 10;19:337–48. doi: 10.1016/j.meegid.2013.04.032 23660484 10.1016/j.meegid.2013.04.032

[13] Guindon S, Dufayard JF, Lefort V, Anisimova M, Hordijk W, Gascuel O. New algorithms and methods to estimate maximum-likelihood phylogenies: assessing the performance of PhyML 3.0. Syst Biol. 2010 5;59(3):307–21. doi: 10.1093/sysbio/syq010 20525638 10.1093/sysbio/syq010

[14] Liu TF, Shafer RW. Web resources for HIV type 1 genotypic-resistance test interpretation. Clin Infect Dis. 2006 6 1;42(11):1608–18. doi: 10.1086/503914 16652319 10.1086/503914 PMC2547473

[15] Vercauteren J, Beheydt G, Prosperi M, Libin P, Imbrechts S, Camacho R, et al Clinical evaluation of Rega 8: an updated genotypic interpretation system that significantly predicts HIV-therapy response. PLoS One. 2013;8(4):e61436 doi: 10.1371/journal.pone.0061436 23613852 10.1371/journal.pone.0061436 PMC3629176

[16] Rhee SY, Jordan MR, Raizes E, Chua A, Parkin N, Kantor R, et al HIV-1 Drug Resistance Mutations: Potential Applications for Point-of-Care Genotypic Resistance Testing. PLoS One. 2015;10(12):e0145772 doi: 10.1371/journal.pone.0145772 26717411 10.1371/journal.pone.0145772 PMC4696791

[17] Gupta RK, Goodall RL, Ranopa M, Kityo C, Munderi P, Lyagoba F, et al High rate of HIV resuppression after viral failure on first-line antiretroviral therapy in the absence of switch to second-line therapy. Clin Infect Dis. 2014 4;58(7):1023–6. doi: 10.1093/cid/cit933 24352348 10.1093/cid/cit933 PMC3952602

[18] Emmett SD, Cunningham CK, Mmbaga BT, Kinabo GD, Schimana W, Swai ME, et al Predicting virologic failure among HIV-1-infected children receiving antiretroviral therapy in Tanzania: a cross-sectional study. J Acquir Immune Defic Syndr. 2010 8;54(4):368–75. doi: 10.1097/QAI.0b013e3181cf4882 20216225 10.1097/QAI.0b013e3181cf4882 PMC4185279

[19] Mgelea EM, Kisenge R, Aboud S. Detecting virological failure in HIV-infected Tanzanian children. S Afr Med J. 2014 10;104(10):696–9. doi: 10.7196/samj.7807 25363057 10.7196/samj.7807

[20] Ferreyra C, Yun O, Eisenberg N, Alonso E, Khamadi AS, Mwau M, et al Evaluation of clinical and immunological markers for predicting virological failure in a HIV/AIDS treatment cohort in Busia, Kenya. PLoS One. 2012;7(11):e49834 doi: 10.1371/journal.pone.0049834 23185450 10.1371/journal.pone.0049834 PMC3504110

[21] Sigaloff KC, Hamers RL, Wallis CL, Kityo C, Siwale M, Ive P, et al Unnecessary antiretroviral treatment switches and accumulation of HIV resistance mutations; two arguments for viral load monitoring in Africa. J Acquir Immune Defic Syndr. 2011 9 1;58(1):23–31. doi: 10.1097/QAI.0b013e318227fc34 21694603 10.1097/QAI.0b013e318227fc34

[22] Salazar-Vizcaya L, Keiser O, Karl T, Davies MA, Haas AD, Blaser N, et al Viral load versus CD4(+) monitoring and 5-year outcomes of antiretroviral therapy in HIV-positive children in Southern Africa: a cohort-based modelling study. AIDS. 2014 10 23;28(16):2451–60. 25392857 10.1097/qad.0000000000000446 PMC4231439

[23] Petersen ML, Tran L, Geng EH, Reynolds SJ, Kambugu A, Wood R, et al Delayed switch of antiretroviral therapy after virologic failure associated with elevated mortality among HIV-infected adults in Africa. AIDS. 2014 9 10;28(14):2097–107. doi: 10.1097/QAD.0000000000000349 24977440 10.1097/QAD.0000000000000349 PMC4317283

